# Supplementary material for: Smartphone-based point-of-care anemia screening in rural Bihar in India
Source: Commun Med (Lond). 2023 Mar 22;3:38. doi: 10.1038/s43856-023-00267-z (PMC10033918; doi:10.1038/s43856-023-00267-z)
Supplement: Supplementary file 1 — Description of Additional Supplementary Files [file 43856_2023_267_MOESM1_ESM.pdf]

Description of Additional Supplementary Files

File Name: Supplementary Data 1

Description: Dataset of clinic-based sample for replicating the analysis.

File Name: Supplementary Data 2

Description: Dataset of pre-school sample for replicating the analysis.

File Name: Supplementary Data 3

Description: Stata code for replicating analysis for clinic-based sample.

File Name: Supplementary Data 4

Description: Stata code for replicating analysis for pre-school sample
