# Supplementary material for: Smartphone-based point-of-care anemia screening in rural Bihar in India
Source: Commun Med (Lond). 2023 Mar 22;3:38. doi: 10.1038/s43856-023-00267-z (PMC10033918; doi:10.1038/s43856-023-00267-z)
Supplement: Supplementary file 2 — Supplementary Materials [file 43856_2023_267_MOESM2_ESM.pdf]

Smartphone-based point-of-care anemia screening in rural Bihar in India

Supplementary Material

1. Supplementary Methods

1.1 Anthropometric characteristics

The following describes how anthropometric characteristics of children were measured and the resulting sample characteristics. The children's age was recorded in full years. Many times, a more precise indication of age in months was not possible since the exact date of birth was not remembered. Assuming that the true age of the children was rather underestimated when stated by parents, the child's age in years was multiplied by 12 months and then 6 months were added. The WHO Child Growth Standards STATA igrowup package and WHO Reference 2007 STATA macro package, provided by the WHO Anthro software, served to compute the following z-scores: weight-for-age z-score (WAZ), height-for-age z-score (HAZ) and weight-for-height z-score (WHZ) for under-fives and WAZ, HAZ and BMI-for-age z-score (BAZ) for children ≥5 years of age. For the group of children and adolescents aged 5-19 years, the WHO Growth Reference (2007) is referred to.[1] Children from birth to the age of 5 years were aligned with the WHO Child Growth Standards.[2] The major types of childhood undernutrition, stunting, underweight, wasting (children <5 years) and thinness (children ≥5 years), were assigned a z-score of below -2 SD from the median of the reference population.[3] The Composite Index of Anthropometric Failure (CIAF) proposed by Svedberg was calculated as an aggregate indicator of undernutrition for under-fives.[4,5] The WHO defines acute malnutrition as MUAC <12.5 cm for children aged 6 to 59 months.[3] The MUAC data of the under-fives were classified according to this categorization and the prevalence of children with acute malnutrition was determined.

High proportions of stunting (39.9%) and underweight (33.7%) were recorded in the children of the pre-school sample. Among under-fives, wasting was discernible in 8.8% and CIAF identified undernutrition in 45.1%. In children aged ≥5 years, 17.2% showed low BMI-for-age (Supplementary Table 1).

1.2 Relationship of nail category with validity of the App

Analyses conducted for the full samples as described in the main manuscript were repeated for the sub-samples of those with nails in categories 1 and 2. The 154 observations of nail categories 1 and 2 in the clinic-based sample showed a very similar distribution of Hb values

in both the App readings and corresponding reference measurements (see Supplementary Table 2, panel a). The p-value of 0.886 when performing the paired t-test indicated that the mean values in this subset did not differ significantly from those of the reference test. The low Pearson correlation coefficient of r=0.242 (p=0.0025) implied a weak positive correlation, which was also visually reflected in the corresponding scatterplot (Supplementary Figure 4, panel a). 95% LoA from -4.21 to 4.16 g/dl were found when performing the Bland-Altman analysis, showing an accuracy of ±4.19 g/dl with a minimal bias of -0.025 g/dl (Supplementary Figure 5, panel a). The average error magnitude hardly changed at 1.70 g/dl (Table 3, panel a). 39% of category 1 and 2 nails fell within the pre-defined range of ±1 g/dl, 73.4% lay within ±2.4 g/dl of the reference test. Moreover, 47.4% of the high quality nails fell within the range of ±10% deviation, whereas only 40.4% of the total nails could be assigned to this range (Table 4, panel a). No considerable improvement was observed in terms of sensitivity, specificity and predictive values (Table 5, panel a). AUC levels of the ROC curves also remained low for both men (0.59) and women (0.53), as shown in Supplementary Figure 6, panels a and b, respectively.

Within the pre-school sample, mean and median Hb values in this subgroup of categories 1 and 2 nails (n=103) were found to be very similar, indicating a symmetrical distribution (Supplementary Table 2, panel b). However, the paired t-test revealed a significant mean difference when comparing with the reference test (t=10.21, p=0.000). The scatterplot (Supplementary Figure 4, panel b) presents a rather diffuse distribution of data points, indicating a weak correlation, which is also expressed by the low Pearson correlation coefficient of r=0.283 (p=0.004). At 95% LoA of -1.46 to 4.54 g/dl and a bias of 1.54 g/dl, an accuracy of ±3.00 g/dl was concluded (Supplementary Figure 5, panel b and Table 3, panel b). Table 4, panel b contains the proportion of observations, which can be associated to the respective deviation ranges. Regarding sensitivity (11.1%) and specificity (96.1%), a high discrepancy between these two statistical figures could be found in high quality nails (Table 5, panel b). The ROC analysis would not be reliable due to the small number of observations and was therefore not performed in this subgroup.

2. Supplementary Tables

Supplementary Table 1: Anthropometric characteristics of pre-school sample

Characteristics	total		boys		girls	
	**n**	**%**	**n**	**%**	**n**	**%**
	**179**	**100**	**81**	**45.3**	**98**	**54.8**
Weight[a]	14.9 ± 2.5		15.0 ± 2.7		14.7 ± 2.3	
(kg, mean±SD, range)	(8.4 - 23.8)		(8.4 - 23.8)		(8.6 - 19.4)	
Height[a]	101.8 ± 9.2		102.2 ± 9.9		101.4 ± 8.6	
(cm, mean±SD, range)	(79.0 - 128.0)		(80.0 - 128.0)		(79.0 - 120.3)	
MUAC[d]	15.1 ± 1.0		15.1 ± 1.1		15.1 ± 0.95	
(cm, mean±SD, range)	(12 - 18)		(12 - 18)		(12.2 - 17)	
HAZ[a]	-1.56		-1.56		-1.54	
(median, IQR)	(-2.51; -0.66)		(-2.72; -0.40)		(-2.36; -0.83)	
WAZ[a]	-1.56		-1.60		-1.52	
(median, IQR)	(-2.27; -0.84)		(-2.38; -0.96)		(-2.21; -0.83)	
WHZ[b]	-0.66		-0.53		-0.85	
(median, IQR)	(-1.19; -0.08)		(-1.27; -0.02)		(-1.08; -0.19)	
BAZ[c]	-1.06		-1.12		-1.04	
(median, IQR)	(-1.78; -0.48)		(-1.81; -0.62)		(-1.69; -0.39)	
Stunting[a]	71	39.9	32	39.5	39	40.2
Underweight[a]	60	33.7	29	35.8	31	32.0
Wasting[b]	8	8.8	4	11.1	4	7.3
Thinness[c]	15	17.2	10	22.2	5	11.9
Acute malnutrition[d]	2	2.2	1	2.8	1	1.9
CIAF[b]	41	45.1	18	50.0	23	41.8

Note: Children's characteristics including weight, height, MUAC, HAZ, WAZ, WHZ, BAZ and nutritional status for the total study population (n=179) and by sex (pre-school sample). MAUC = mid-upper arm circumference, HAZ = heigt-for-age z-score, WAZ = weight-for-age z-score, WHZ = weight-for-height z-score, BAZ = BMI (body mass index)-for-age z-score, CIAF = Composite Index of Anthropometric Failure, SD = standard deviation, IQR = inter-quartile range. WHO Child Growth Standards STATA igrowup package (for children <5 years of age) and WHO Reference 2007 STATA macro package (for children ≥5 years of age) were used to compute z-scores. [a] n=178 (male: n=81, female: n=97); [b] children <60 months of age, n=91 (male: n=36, female: n=55); [c] children ≥60 months of age, n=87 (male: n=45, female: n=42); [d] children <60 months of age, n=90 (male: n=36, female: n=54).

Supplementary Table 2: Descriptive statistics of hemoglobin measurements

	Method of measurement	n	Mean	SD	Median	IQR	Min	Max	t-value	r
**a**	Automated hematology analyzer	272	12.58	1.88	12.6	2.5	3.1	17.1		
	Sanguina Smartphone App	272	12.20	1.75	12.2	2.4	7.2	16.3	-2.77*	0.225*
	Automated hematology analyzer (subgroup nail category 1 and 2)	154	12.61	1.88	12.6	2.4	3.1	17.1		
	Sanguina Smartphone App (nail category 1 and 2)	154	12.59	1.56	12.6	2.1	7.3	15.7	-0.14	0.242*
**b**	HemoCue Hb 301 photometer	179	11.79	1.11	11.9	1.3	7.3	14.6		
	Sanguina Smartphone App	179	13.09	1.71	13.2	1.8	4.0	18.6	9.67*	0.235*
	HemoCue Hb 301 photometer (subgroup nail category 1 and 2)	103	11.77	1.21	11.9	1.5	7.3	14.6		
	Sanguina Smartphone App (nail category 1 and 2)	103	13.31	1.34	13.4	1.6	8.6	15.7	10.21*	0.283*
**c**	HemoCue Hb 301 photometer	160	11.77	1.13	11.9	1.4	7.3	14.6		
	Sanguina Smartphone App retrained	160	12.02	0.67	12.1	0.7	9.2	14.7	2.74*	0.265*

Note: Distribution of hemoglobin concentrations (g/dl) measured by the respective method; SD = standard deviation; IQR = inter-quartile range; t-value of pairwise comparison of means (reference vs. App) using paired t-test; Pearson correlation coefficient r; *p≤0.05 indicates statistical significance; **a** clinic-based sample, **b** pre-school sample, **c** after App retraining.

Supplementary Table 3: Repeatability in clinic-based sample

Measurement	Mean	SD	95% CI
Right hand, n=185			
1st	12.24	1.69	12.0; 12.5
2nd	12.18	1.68	11.9; 12.4
3rd	12.15	1.66	11.9; 12.4
4th	12.14	1.59	11.9; 12.4
5th	12.09	1.65	11.8; 12.3
Left hand, n=80			
1st	12.51	1.68	12.1; 12.9
2nd	12.37	1.69	12.0; 12.7
3rd	12.25	1.71	11.9; 12.6
4th	12.35	1.75	12.0; 12.7
5th	12.22	1.76	11.8; 12.6

Note: Descriptive statistics (mean, standard deviation (SD), 95% Confidence Interval (CI)) in g/dl for the 5 repeated App hemoglobin measurements of the right and left hand (clinic-based sample).

Supplementary Table 4: Repeatability in pre-school sample

Measurement (n=162)	Mean	SD	95% CI
1st	13.08	1.74	12.8; 13.4
2nd	13.12	1.91	12.8; 13.4
3rd	13.12	1.64	12.9; 13.4

Note: Descriptive statistics (mean, standard deviation (SD), 95% Confidence Interval (CI)) in g/dl for the 3 repeated App hemoglobin measurements of the right and left hand (pre-school sample).

3. Supplementary Figures

Supplementary Figure 1: Selection of clinic-based sample

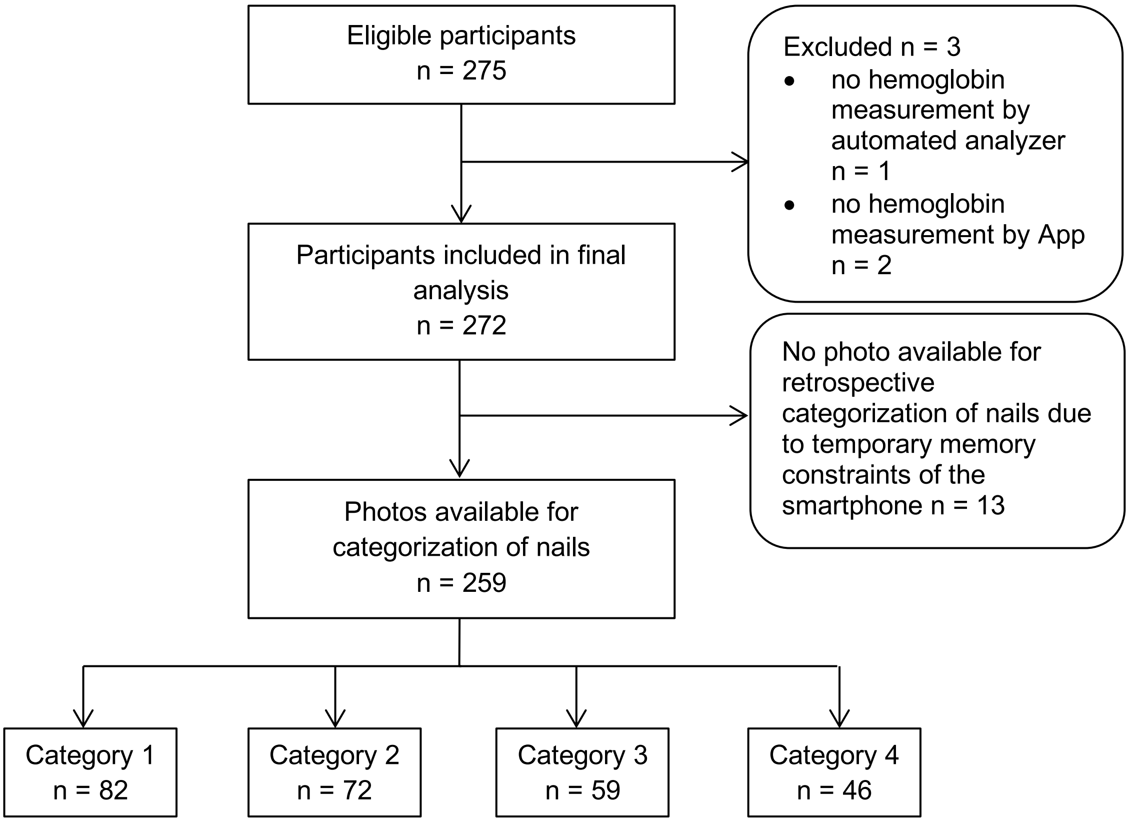

Note: Flow chart of recruitment and inclusion of study participants of the clinic-based sample. Source data is in Supplementary Data 1.

Supplementary Figure 2: Selection of pre-school sample

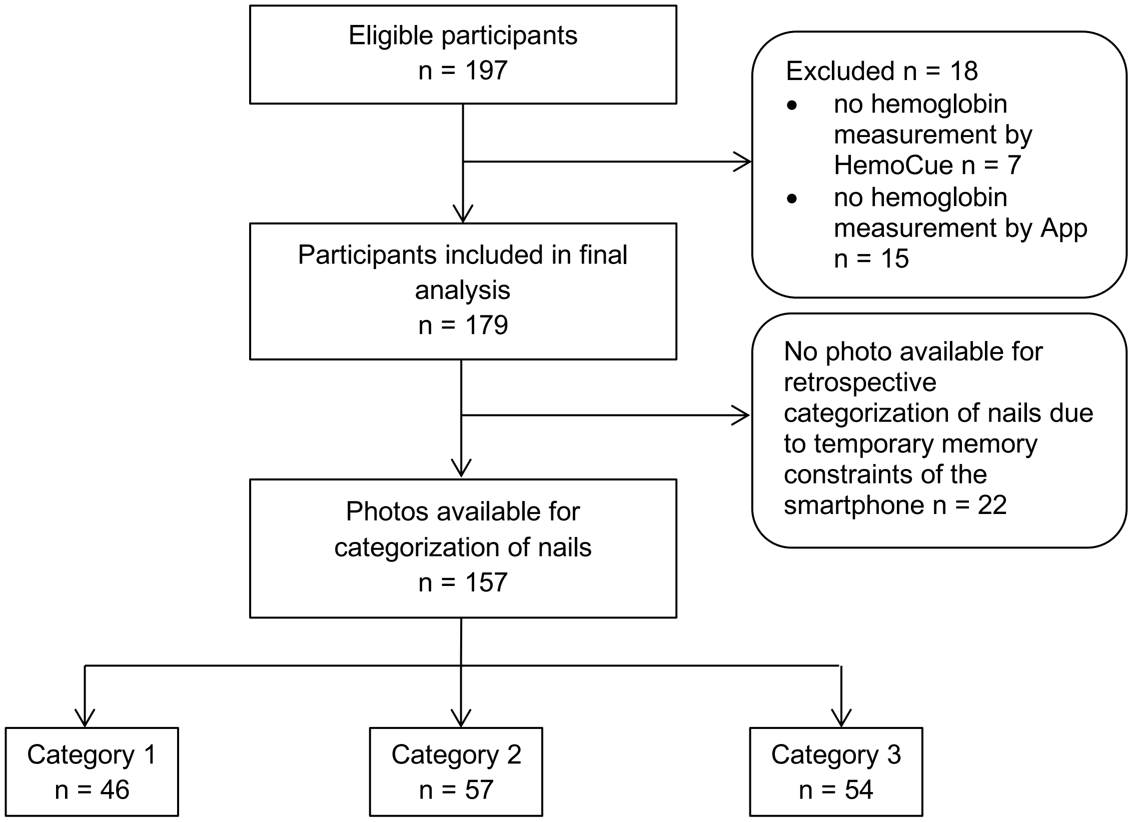

Note: Flow chart of recruitment and inclusion of study participants of the pre-school sample. Source data is in Supplementary Data 2.

Supplementary Figure 3: Sample selection for anthropometric analysis

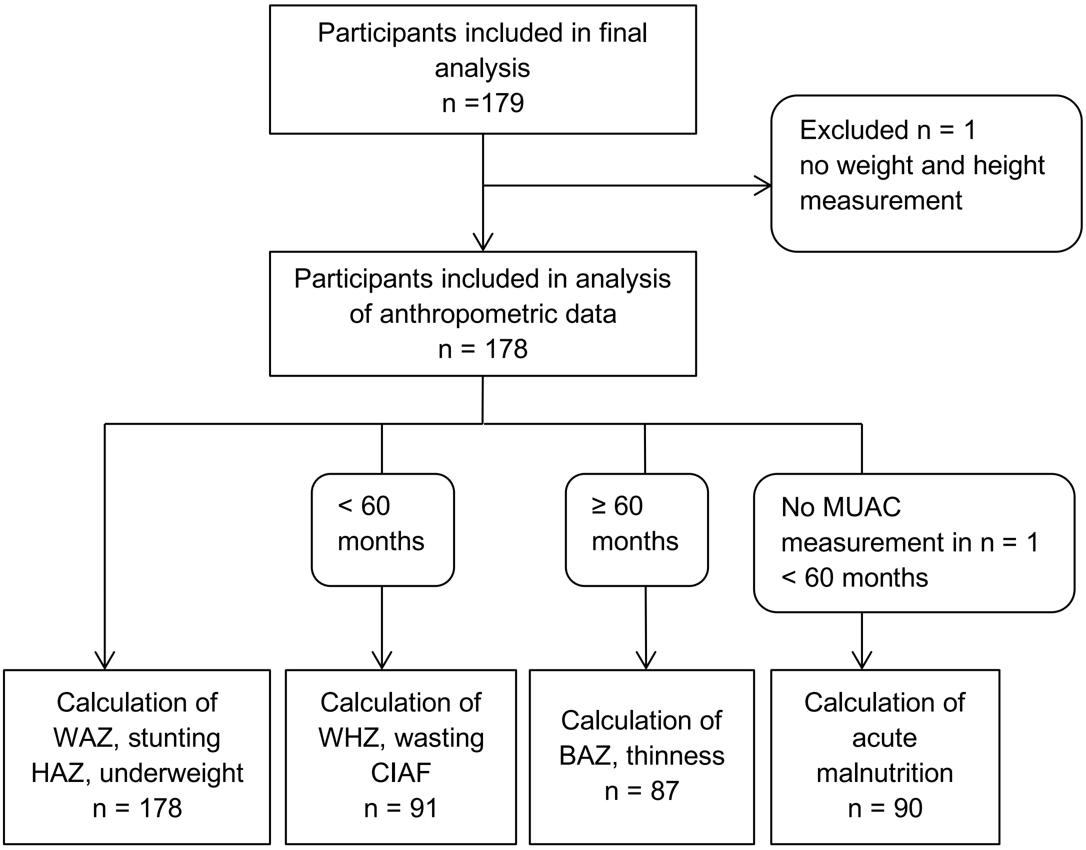

Note: Flow chart of inclusion of study participants of the pre-school sample for analysis of anthropometric data. Source data is in Supplementary Data 2.

Supplementary Figure 4: Hemoglobin measurements by App and reference method for nail categories 1 and 2

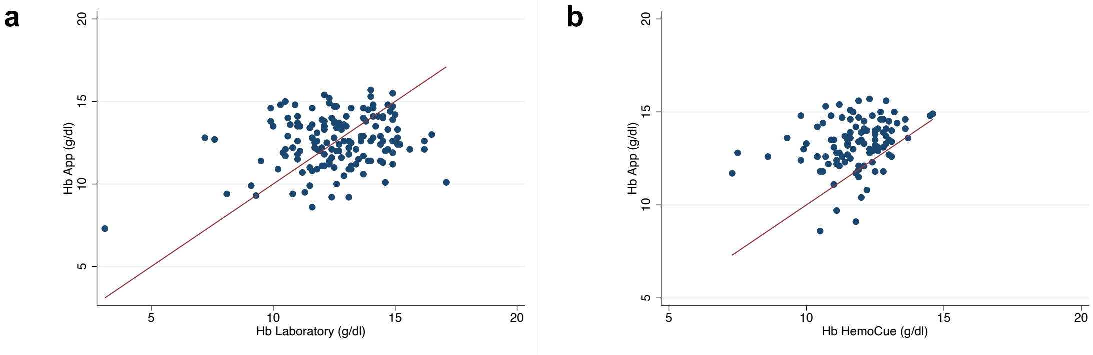

Note: Scatterplot showing App hemoglobin (Hb) measurements of nail categories 1 and 2 (y-axis) plotted against **a** the laboratory reference (x-axis) in the clinic-based sample (n=154) and **b** the HemoCue reference in the pre-school sample (n=103). The red diagonal line represents the line of equality where App Hb concentration is equal to the respective reference Hb concentration. Source data for panel **a** is in Supplementary Data 1; source data for panel **b** is in Supplementary Data 2.

Supplementary Figure 5: Bland-Altman plot for nail categories 1 and 2

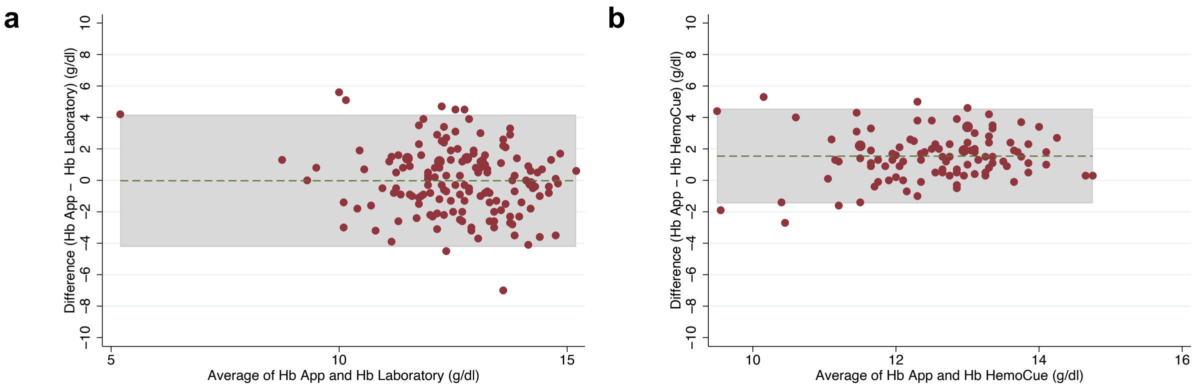

Note: Bland-Altman plot. The green dashed line represents the mean difference (bias), the gray shaded area represents the 95% limits of agreement (LoA), determined as bias ±1.96 SD; **a** for clinic-based sample, difference (Hb App (nail category 1 and 2) – Hb Laboratory) on y-axis, average of Hb App (nail categories 1 and 2) and Hb Laboratory on x-axis, bias = -0.025 g/dl, LoA -4.21, 4.16 (n=154); **b** for pre-school sample, difference (Hb App (nail categories 1 and 2) – Hb HemoCue) on y-axis, average of Hb App (nail categories 1 and 2) and Hb HemoCue on x-axis, bias = 1.54 g/dl, LoA = -1.46, 4.54 (n=103). Source data for panel **a** is in Supplementary Data 1; source data for panel **b** is in Supplementary Data 2.

Supplementary Figure 6: Receiver-operating characteristic (ROC) curve for nail categories 1 and 2, clinic-based sample

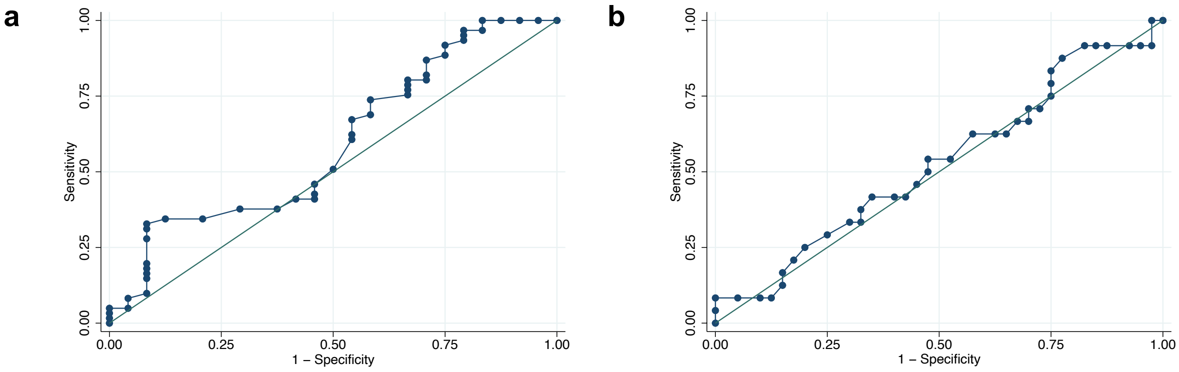

Note: Receiver-operating characteristic (ROC) curve with an area under the curve (AUC) of **a** 0.59 for men (≥15 years of age, n=85) and **b** 0.53 for women (≥15 years of age, n=64) based on App hemoglobin measurements of nail categories 1 and 2 in the clinic-based sample. Source data is in Supplementary Data 1.

Supplementary References

1. Onis M de, Onyango AW, Borghi E, Siyam A, Nishida C, Siekmann J. Development of a WHO growth reference for school-aged children and adolescents. *Bulletin of the World health Organization*. 2007;85(9):660-667.

2. World Health Organization. *WHO Child Growth Standards: Length/Height-for-Age, Weight-for-Age, Weight-for-Length, Weight-for-Height and Body Mass Index-for-Age: Methods and Development*. World Health Organization; 2006. https://www.who.int/publications/i/item/924154693X

3. Cashin K, Oot L. *Guide to Anthropometry: A Practical Tool for Program Planners, Managers, and Implementers*. Planners, Managers, and Implementers. Washington, DC: Food and Nutrition Technical Assistance III Project (FANTA)/ FHI 360; 2018. https://www.fantaproject.org/sites/default/files/resources/FANTA-Anthropometry-Guide-May2018.pdf

4. Nandy S, Irving M, Gordon D, Subramanian SV, Smith GD. Poverty, child undernutrition and morbidity: new evidence from India. *Bulletin of the World Health Organization*. 2005;83:210-216.

5. Svedberg P. *Poverty and Undernutrition: Theory, Measurement, and Policy*. Oxford University Press; 2000.
