# Supplementary material for: Smartphone-based point-of-care anemia screening in rural Bihar in India
Source: Commun Med (Lond). 2023 Mar 22;3:38. doi: 10.1038/s43856-023-00267-z (PMC10033918; doi:10.1038/s43856-023-00267-z)
Supplement: Supplementary file 8 — Reporting Summary [file 43856_2023_267_MOESM8_ESM.pdf]

nature research

Corresponding author(s): Lisa Bogler

Last updated by author(s): Feb 2, 2023

Reporting Summary

Nature Research wishes to improve the reproducibility of the work that we publish. This form provides structure for consistency and transparency in reporting. For further information on Nature Research policies, see our Editorial Policies and the Editorial Policy Checklist.

Statistics

For all statistical analyses, confirm that the following items are present in the figure legend, table legend, main text, or Methods section.

n/a	Confirmed	
☐	☒	The exact sample size (*n*) for each experimental group/condition, given as a discrete number and unit of measurement
☐	☒	A statement on whether measurements were taken from distinct samples or whether the same sample was measured repeatedly
☐	☒	The statistical test(s) used AND whether they are one- or two-sided<br>*Only common tests should be described solely by name; describe more complex techniques in the Methods section.*
☐	☒	A description of all covariates tested
☐	☒	A description of any assumptions or corrections, such as tests of normality and adjustment for multiple comparisons
☐	☒	A full description of the statistical parameters including central tendency (e.g. means) or other basic estimates (e.g. regression coefficient) AND variation (e.g. standard deviation) or associated estimates of uncertainty (e.g. confidence intervals)
☐	☒	For null hypothesis testing, the test statistic (e.g. *F*, *t*, *r*) with confidence intervals, effect sizes, degrees of freedom and *P* value noted<br>*Give P values as exact values whenever suitable.*
☒	☐	For Bayesian analysis, information on the choice of priors and Markov chain Monte Carlo settings
☒	☐	For hierarchical and complex designs, identification of the appropriate level for tests and full reporting of outcomes
☐	☒	Estimates of effect sizes (e.g. Cohen's *d*, Pearson's *r*), indicating how they were calculated

Our web collection on statistics for biologists contains articles on many of the points above.

Software and code

Policy information about availability of computer code

Data collection	The Sanguina Smartphone App for hemoglobin measurement, version of November 2019 as provided by developer, was used to collect hemoglobin measurements.
Data analysis	Stata SE 15 was used for data analysis.

For manuscripts utilizing custom algorithms or software that are central to the research but not yet described in published literature, software must be made available to editors and reviewers. We strongly encourage code deposition in a community repository (e.g. GitHub). See the Nature Research guidelines for submitting code & software for further information.

Data

Policy information about availability of data

All manuscripts must include a data availability statement. This statement should provide the following information, where applicable:

- Accession codes, unique identifiers, or web links for publicly available datasets
- A list of figures that have associated raw data
- A description of any restrictions on data availability

All data generated or analysed during this study are included in this published article and its supplementary information files.

Field-specific reporting

Please select the one below that is the best fit for your research. If you are not sure, read the appropriate sections before making your selection.

☐ Life sciences ☒ Behavioural & social sciences ☐ Ecological, evolutionary & environmental sciences

For a reference copy of the document with all sections, see nature.com/documents/nr-reporting-summary-flat.pdf

Behavioural & social sciences study design

All studies must disclose on these points even when the disclosure is negative.

Study description	This study is a quantitative validation study of a hemoglobin measurement tool in two cross-sectional samples.
Research sample	Sample 1 consists of outpatient attendees (mean age 33.5 years, 41% female) in a community-based clinic in Madhepura City, Bihar, India. They were either patients who were referred for a hematological investigation or they accompanied family members. It was therefore a sample where harm from drawing blood was minimised as most patients were meant to have a blood test done irrespective of study participation. Other patients, who were not scheduled to have their blood drawn, as well as family members who accompanied patients to the clinic, requested to benefit from knowing their hemoglobin value and volunteered to participate in the study. In addition, close cooperation with a laboratory for blood analysis was feasible in this sample.<br>Sample 2 consists of children aged 2 to 7 years attending five pre-schools in villages in Madhepura district, Bihar, India. This sample was chosen because a related study planned to use the app for hemoglobin measurement among children attending pre-schools in Madhepura if this validation study found sufficient accuracy.
Sampling strategy	Sample 1: Convenience sample. All individuals coming to the clinic who were willing to participate were included.<br>Sample 2: Convenience sample. All children attending the pre-schools on the day or called to participate in the data collection by the care taker of the pre-schools were included, if the parent or guardian agreed.<br>A sample size calculation was performed. The sample size was chosen to be sufficiently large to identify the accuracy as reported in the original study.
Data collection	Hemoglobin was measured with the Sanguina Smartphone App on an iPhone 5s, by a trained member of the research team. In Sample 1, venous blood sample was analysed by the Aspen Mindray BC-500. The blood sample was drawn by a trained laboratory assistant. In Sample 2, a blood drop was analysed using the HemoCue Hb 301 device. This blood drop was taken by a medical doctor. In both samples, sociodemographic questions were recorded on paper by trained research assistants. In Sample 2, weight, height, and mid-upper arm circumference measurements were additionally recorded on paper and measured by trained research assistants using a digital scale, stadiometer, and measuring tape. Apart from individuals directly involved in data collection (participants, enumerators), in Sample 2, the caretaker and parents were present during data collection. All research assistants and the laboratory assistant were blind to the study hypothesis.
Timing	Data collection took place between December 2019 and January 2020.
Data exclusions	Data was excluded from analysis if information was missing for any measurement. From the main analysis of validity, 3 observations were excluded in Sample 1, 18 observations were excluded in Sample 2. Nail categories could only be determined for observations where pictures were still available, leading to exclusions of 13 and 22 observations in the Sample 1 and 2, respectively, from the analysis of nail categories.
Non-participation	In Sample 1, no participant dropped out or declined participation. In Sample 2, some children present at the pre-school did not participate due to time restrictions (hemoglobin was not measured by either App or HemoCue).
Randomization	Participants were not randomly assigned to groups, because the validation study did not include experimental groups.

Reporting for specific materials, systems and methods

We require information from authors about some types of materials, experimental systems and methods used in many studies. Here, indicate whether each material, system or method listed is relevant to your study. If you are not sure if a list item applies to your research, read the appropriate section before selecting a response.

Materials & experimental systems

n/a	Involved in the study	
☒	☐	Antibodies
☒	☐	Eukaryotic cell lines
☒	☐	Palaeontology and archaeology
☒	☐	Animals and other organisms
☐	☒	Human research participants
☒	☐	Clinical data
☒	☐	Dual use research of concern

Methods

n/a	Involved in the study	
☒	☐	ChIP-seq
☒	☐	Flow cytometry
☒	☐	MRI-based neuroimaging

Human research participants

Policy information about studies involving human research participants

Population characteristics	See above.
Recruitment	Sample 1: Participants were either patients of the community-based clinic who were referred for a hematological investigation or they accompanied family members. The sample is therefore not representative of the general population. Population not attending such a clinic is not represented in this study - for example more wealthy individuals or currently healthy. As we do not assess prevalence of any disease, this does not influence the analysis of the validity of the app.<br>Sample 2: Participants were children of pre-schools (Anganwadi Centres) and are representative of children attending other pre-schools (Anganwadi Centres) in this district. All children of the pre-schools whose parents agreed to be included and who were present were allowed to participate.
Ethics oversight	The study protocol was reviewed by the Ethics Committee of the University of Göttingen, Germany and received a statement of no objection.

Note that full information on the approval of the study protocol must also be provided in the manuscript.
